# Severe acute polyradiculoneuritis revealing systemic lupus erythematosus: a case report

**DOI:** 10.11604/pamj.2023.45.8.32236

**Published:** 2023-05-04

**Authors:** Assma Boudanga, Mohamed Chraa, Oussama Cherkaoui Rhazouani, Najib Kissani

**Affiliations:** 1 Department of Neurology, University Teaching Hospital Mohammed VI, Marrakech, Morocco,; 2 Moroccan Society of Neurophysiology, Marrakech, Morocco,; 3 Laboratory of Physiology, Faculty of Medicine and Pharmacy of Marrakech, Marrakech, Morocco,; 4 Laboratory of Clinical and Experimental Neuroscience, Faculty of Medicine, Cadi Ayyad University Marrakech, Marrakech, Morocco

**Keywords:** Acute polyradiculoneuropathy, systemic lupus erythematosus, peripheral neuropathy, Guillain-Barré, case report

## Abstract

Systemic Lupus Erythematosus (SLE) is a common disease with extremely heterogeneous neurological manifestations in its clinical expression. However, few cases have been reported in the last 50 years when the initial manifestation of SLE is Guillain-Barré syndrome (GBS). Our work highlights the importance of evoking SLE as a potential etiology in a patient presenting with acute polyradiculoneuritis. We report the case of a 41-year-old woman who presented with dyspnoea with a purely proxo-distal motor deficit in all four limbs with dermatological lesions such as generalized myxedema and alopecia. The clinical electrical and biological presentation confirms acute polyradiculoneuritis revealing systemic lupus erythematosus. The outcome was marked by clinical improvement, despite the severity of the clinical picture, after treatment with corticosteroid and cyclophosphamide boluses. In conclusion, neurological manifestations in lupus disease are common, whereas the form of acute polyradiculoneuropathy is very rare with a committed vital prognosis. Early diagnosis and management are essential.

## Introduction

The neurological manifestations of systemic lupus erythematosus (SLE) are frequent but extremely heterogeneous in their clinical expression. Depending on the criteria used, peripheral nervous system involvement is not significant and varies from 2% to 30% [1]. Multiple mononeuropathies, including axonal, distal and symmetrical, sensory or sensori-motor polyneuropathies, or cranial nerve involvement are common [2]. However, few cases have been reported in the last 50 years where the initial manifestation of SLE is Guillain-Barré syndrome (GBS). The first case was confirmed in 1964 by Gargour *et al*. [3]. Our work highlights the importance of evoking SLE as a potential etiology in a patient presenting with acute polyradiculoneuritis.

## Patient and observation

**Patient information:** a 41-year-old woman with a history of three miscarriages and skin photosensitivity, without other medical, family, psycho-social history or past interventions, who presented with a symptomatology of abrupt onset, admitted in the plateau phase, made up of a synchronous and symmetrical ascending functional impotence of both lower limbs 20 days before admission.

**Figure 1:**
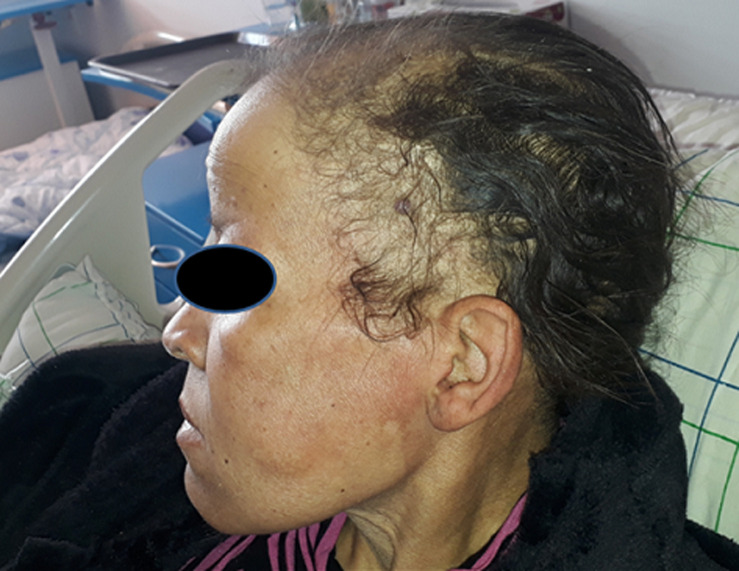
alopecia

**Clinical findings:** the clinical examination found a dyspneic patient with a motor deficit evaluated at 4/5 proximal and 3/5 distal to the four limbs, a hypotonia more accentuated in the two lower limbs and an osteotendinous areflexia in the four limbs. Sensibility and cranial pairs were intact with a plantar skin reflex in flexion. Dermatological examination revealed generalized myxedema associated with alopecia (Figure 1).

**Figure 2:**
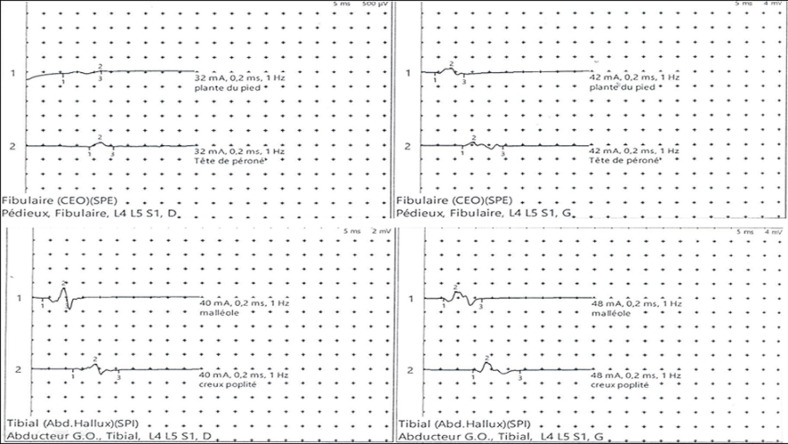
collapse of motor action potential in the lower limbs

**Diagnostic assessment:** electroneuromyography (ENMG) revealed collapse of motor potential and absence of F waves in all four limbs. The study of the sensory nerve conduction is without abnormality suggesting an acute axonal motor polyradiculoneuropathy more marked in the two lower limbs (Figure 2). Biological abnormalities were in favor of SLE (Table 1). Radiological exploration revealed hepatomegaly and large effusions: peritoneal, pericardial, and bilateral pleural, which explain the severity of the clinical picture. Complete brain and spinal cord magnetic resonance imaging (MRI) were normal as part of the extension workup.

**Table 1:** the results of the biological assessment

Biological assessment	Result
Cerebrospinal fluid	Appearance of clear liquid, with slight hyperproteinorache at 0.50g/L, glycorachy at 0.5g/L and normal cellulorachy
Complete blood count	The complete blood count showed bicytopenia with normochromic normocytic anemia (hemoglobin level at 7.1 g/dL, mean corpuscular volume at 82.2 fL, mean corpuscular hemoglobin concentration at 33.3 g/dL and teneur corpusculaire moyenne en hémoglobin at 27.4 pg) and lymphopenia at 2970 elements/mm^3^ without thrombocytopenia (platelets at 293,000/mm^3^)
Sedimentation rate	the sedimentation rate is accelerated up to 60 mm in the first hour
C reactive protein	C-reactive protein increased to 15.05 mg/l
Albuminemia	hypoalbuminemia at 10.3 g/L
Protidemie	hypoprotidemia at 54.4 g/L
Proteinuria	Proteinuria was 4.83 g/24 hours
Serologies	Syphilitic serology showed a positive treponema pallidum haemagglutination of 1280 and a negative Venereal disease research laboratories; Hepatitis B, C and HIV serologies were negative
Immunological assessment	Immunoassays revealed positive ANA with a speckled pattern titer of 1/1280, anti-dsDNA antibody greater than 300 IU/mL (normal <20), C3 and C4 were normal

**Diagnostic/therapeutic intervention:** the diagnosis of acute Guillain-Barré type Pro Re Nata (PRN) associated with lupus disease was retained, and a bolus of methyl-prednisolone at a dose of 1g per day was established for five days. The patient benefited from monthly courses of cyclophosphamide 1g, synthetic antimalarials such as hydroxychloroquine (Plaquenil) 400 mg per day, and complementary corticosteroid therapy of 60 mg for six weeks with progressive reduction of 5 mg every 15 days.

**Follow-up and outcomes:** the evolution is marked by a clear clinical improvement with recovery of walking, a markedly improved muscle testing and no side effects related to the treatment. The prognosis of our case is clearly good explained by the therapeutic precocity.

**The patient’s perspective:** the patient reports satisfaction following the recovery of motor functions and her autonomy with a marked improvement in her quality of life. The psychological interview of the patient also demonstrates a stable post-therapeutic psychic state.

## Discussion

Systemic lupus is retained for the patient according to the 1982 American College of Rheumatology (ACR) criteria, revised in 1997 and the EULAR/ACR 2019 classification criteria [4]. Peripheral neuropathies are only considered in the list of neuropsychological manifestations in the Systemic Lupus International Collaborating Clinics (SLICC) classification criteria for systemic lupus, which are central, peripheral, autonomic nervous system, and psychiatric syndromes. Peripheral involvement is exceptionally revealing of the disease. Symmetrical distal polyneuropathies, pure sensory or sensory-motor polyneuropathies and multiple mononeuropathies are the most common peripheral disorders [1]. Subacute or chronic PRN characterized by the presence of an inflammatory syndrome and hyperproteinorachia are rare [5], but GBS in its severe form is much rarer. According to prospective studies based on electrophysiological data rather than symptoms, acute PRN can take several forms, the most common of which is sensorimotor axonal neuropathy [6]. In the light of the literature, Hess *et al*. reported in 1990 the first case of Miller Fisher syndrome, a variant of Guillain-Barré, complicating systemic lupus erythematosus with improvement by five sessions of plasmapheresis after therapeutic failure of immunoglobulins. This result is confirmed by the study of Bingisser *et al*. in 1994 [7]. Ait Benhaddou *et al*. 2003, reported a case of acute sensitive motor axonal PRN with SLE, while Quaid Nadri *et al*. 2015, presented the case of a young woman with GBS who was later diagnosed with SLE and class V lupus nephritis (LN) [5,8]. In the case of FZ Haounou *et al*. 2014, it was a demyelinating disease with axonal loss [2]. The other cases described in the literature are mainly demyelinating forms, however in our observation; it is a pure motor axonal involvement.

Like our observation, the cerebrospinal fluid (CSF) study showed no abnormality in most of the other cases (Table 2). The evolution of the patient, in our investigation, is marked by the improvement of neurological signs after the first courses of cyclophosphamide 1g preceded by five boluses of 1g of methylprednisolone. Furthermore, Santiago-Casas Y *et al*. 2013, report the observation of two cases of acute axonal neuropathies of GBS type, the first with acute motor axonal neuropathy, the second with acute motor and sensory axonal neuropathy in the context of SLE. The first case did not respond to treatment with intravenous immunoglobulin (IVIG) and plasmapheresis, whereas the second case progressed favorably after administration of high-dose glucocorticoids and monthly infusions of low-dose intravenous cyclophosphamide. Both patients remained in clinical remission and without neurological sequelae after 10 and 3 years of follow-up, respectively [9]. Furthermore, Quaid Nadri *et al*. 2015 noted the disappearance of neurological symptoms after plasmapheresis, IVIG, and corticosteroid treatment. Cyclophosphamide was not administered, although others have reported success when used [8]. The diagnostic and therapeutic management of neurolupus is a challenge for the clinician. It is still poorly codified and largely determined by the experience of the teams [10]. It is difficult to predict the prognosis. Although a favorable outcome is usual, mortality is not zero, especially in severe forms, including acute PRN with respiratory involvement, where immunosuppressant, plasma exchange and IVIG are often required [5].

**Table 2:** cases of acute polyradiculoneuritis in the context of systemic lupus erythematosus published in the literature

	Ahbeddou N *et al*. 2010	Santiago Casas Y 2013	Ha-ou-nou FZ *et al*. 2014	Quaid Nadri *et al*. 2015	Our case
Sex	F	F	M	F	F
Age	30 years	20 years	44 years	23 years	41 years
Electromyoneurography	Polyradiculoneuritis with sensory-motor disorders of the axonal type	Acute motor axonal neuropathy	Demyelinating and axonal polyradiculoneuropathy	Demyelinating polyneuropathy	Axonal motor polyradiculoneuropathy
Cerebrospinal fluid	Three white blood cells/mm^3^, 0.45 g/l albumin and 0.50 g/l glucose	Normal	Normal		Hyperproteinorachia at 0.50 g/l, glycorachia at 0.5 g/l and normal cytology
AAN AAD	+ 1/640 + 1/160	+ 1:320 +	+ 1/1 280 + 1/640	+ 1/1280 28.2 U/mL	+ 1/1280 + 300 IU/mL
Treatment	Intravenous immunoglobulin at 0.4 g/kg per day and a bolus of methylprednisolone 1 g/d for five days, then relay orally with prednisone (60 mg/d)	Intravenous immunoglobulins (IVIG) 0.4 g/kg/day for five days	A bolus of methylprednisolone of 1 g/d for three days was initiated, associated with a course of intravenous immunoglobulins (IVIg) at a dose of 400 mg/kg per day for five days, started on the second day	Methylprednisolone 1 g daily for 3 days plus 9 cycles of plasmapheresis and IVIG for a total of 2 g/kg	A bolus of methylprednisolone 1 g per day for five days followed by courses of cyclophosphamide 1 g per month, synthetic antimalarials such as hydroxychloroquine (Plaquenil) 400 mg per day and additional corticosteroid therapy of 60 mg for six weeks with a gradual reduction of 5 mg every 15 days
Evolution	Rapid improvement of clinical signs, with maintenance of a slight left crural deficit	Persistence of neurological deficits	Unfavorable	Clinical improvement	Clinical improvement

## Conclusion

Acute PRN remains a rare neurological manifestation in SLE. Bolus methylprednisolone and monthly courses of cyclophosphamide are proposed in this clinical presentation, in which a favorable clinical response was obtained despite the severity of the picture, which explains that early management of patients can improve the prognosis of the disease.
